# Population Pharmacokinetics of Danofloxacin in Yellow River Carp (*Cyprinus carpio haematopterus*) After One Single Oral Dose

**DOI:** 10.3389/fvets.2022.868966

**Published:** 2022-04-06

**Authors:** Zhe-Wen Song, Fang Yang, Yan Dai, Chao-Shuo Zhang, Hao-Tian Shao, Han Wang, Kai-Li Ma, Ze-En Li, Fan Yang

**Affiliations:** Department of Veterinary Pharmacology, College of Animal Science and Technology, Henan University of Science and Technology, Luoyang, China

**Keywords:** population pharmacokinetics, danofloxacin, Yellow River carp, sparse sampling, oral dosing

## Abstract

This study aimed to determine the population pharmacokinetics of danofloxacin in healthy Yellow River carp (*Cyprinus carpio Haematopterus*) after single oral administration at 10 mg/kg body weight (BW). A sparse sampling was applied in this study and plasma samples were randomly collected from the tail veins of six carp at 0.25, 0.5, 1, 2, 4, 6, 8, 12, 16, 24, 36, 48, 72, 96, 120 and 144 h after administration. A maximum of four plasma samples was collected from each carp. Then the concentrations of danofloxacin in plasma samples were determined through an HPLC method. Danofloxacin could be quantified in plasma up to 144 h after administration. The corresponding population pharmacokinetic modeling was developed according to the non-linear mixed effect method, including covariate and covariance models to explain some variations from unknown sources and improve the prediction ability. On the premise of sparse sampling, the typical values of the population (fixed effect) and inter-individual variation (random effect) were described by the current population pharmacokinetic model. The estimated typical values and coefficient of variation between individuals (CV%) of absorption rate constant (tvKa), apparent distribution volume (tvV) and clearance (tvCL) were 2.48 h^−1^ and 0.203%, 47.8 L/kg and 8.40%, 0.694 L/h/kg and 4.35%, respectively. The current danofloxacin oral dosing (10 mg/kg BW) can provide suitable plasma concentrations to inhibit those pathogens with MIC values below 0.016 μg/ml based on the calculated PK/PD indices of AUC/MIC or C_max_/MIC. Further studies are still needed to determine the *in vitro* and *in vivo* antibacterial efficacy of danofloxacin against pathogens isolated from Yellow River carp and finally draw a reasonable dosing regimen.

## Introduction

Common carp (*Cyprinus carpio*) is often cultivated in Asia with solid adaptability, excellent reproductive ability, and rich nutritional value. Especially in China, it has become one of the most commonly farmed fish species. Yellow River carp (*Cyprinus carpio Haematopterus*) is a valuable variety with the crucial economic value among different carp species (1). It mainly referred to the carp living in the Yellow River. However, they have also been artificially cultivated nationwide in a wide temperature range of 5 to 28°C. Yellow River carp have stronger disease resistance and more delicious meat than the common carp (2).

Danofloxacin is a fluoroquinolone antibacterial developed exclusively for veterinary medicine in the 1990's (3, 4), and it acts by inhibiting bacterial DNA-gyrase. Because of its broad antimicrobial spectrum, danofloxacin has been licensed in bovine, ovine, caprine, and poultry species (5). With a low minimum inhibitory concentration (MIC) value against most aquatic germs (6, 7), danofloxacin is also used to treat skin infections and septicemia in fish (8). In European Union, danofloxacin has been approved in fish species by the Commission Regulation (EU) No 37/2010 (9). However, danofloxacin is now used in China extra-label to treat fish diseases, and the dosage regimen is always extrapolated from other mammalian species. As poikilotherms, however, fish are physiologically and anatomically different from mammals. Therefore, the dosing regimen for mammals may not be suitable for fish. Fluoroquinolones are critically important antimicrobials for human medicine; therefore, more prudent use of danofloxacin in aquaculture should be advocated to minimize the development of resistance, and the knowledge about the pharmacokinetic and rational selection of dosage regimes will promote its prudent use.

The pharmacokinetics of danofloxacin has been reported in some fish species, including amur sturgeon (10), rainbow trout (11), brown trout (6), African catfish (12), and ornamental fish (koi) (13), which showed a long elimination half-life, high bioavailability, and good tissue penetration. However, significant pharmacokinetic differences were also exhibited among different fish species, and the extravascular bioavailability varied from 67.12% in catfish (12) to 105.87% in rainbow trout (11).

Unlike other mammals or poultry, most fish are small in size and have limited blood flow. Based on animal welfare considerations, continuous blood sampling from one fish is not possible. Therefore, in the standard pharmacokinetic study conducted in fish, blood is typically collected from 5 to 6 fish at each time point, and each fish would be only collected once. Then the average concentrations vs. time data are used to determine the pharmacokinetic parameters. The traditional sampling method requires more animals and more time. In addition, the conventional results can not reflect the differences among individuals (14, 15). Conversely, the population pharmacokinetic analysis combined with a sparse sampling method can solve these limitations and clarify the variations in the populations (16). Therefore, the current study aimed to determine (I) the population pharmacokinetics of danofloxacin in Yellow River carp after single oral administration at 10 mg/kg body weight (BW) based on a sparse sampling method and non-linear mixed effect modeling and (II) the values of PK/PD parameters (AUC/MIC and C_max_/MIC) using recent MIC data for susceptible pathogens and pharmacokinetic parameters obtained in this study.

## Materials and Methods

### Chemical Reagents

The analytical standard for danofloxacin mesylate (Lot No. h0201210) with a purity of 94.2% was purchased from the China Institute of Veterinary Drugs Control (Beijing, China). The raw material of danofloxacin mesylate (Lot No. 201217–1) with a purity of 95.37% was donated by Zhejiang Guobang Pharmaceutical Co., Ltd. (Hangzhou, China). Acetonitrile and methanol were of HPLC grade and supplied by Xilong Chemical Co., Ltd. (Shantou, China). Triethylamine was a domestic analytical reagent purchased from Tianjin Kemi O Chemical Reagent Co., Ltd. (Tianjin, China).

### Animals

Thirty healthy Yellow River carp (*Cyprinus carpio Haematopterus*) were purchased from Mianchi Qinglianhe Aquaculture Co., Ltd. (Sanmenxia, China). Their average BW was 0.38 kg (0.25–0.54 kg). Twenty-four fish in the population were equally divided into four groups, and the other 6 fish served as a control group to supply blank plasma samples. Each group was reared in a cuboid tank (1.3 m × 0.8 m × 0.65 m, Length × width × height) under continuous aeration. The water was analyzed daily for quality parameters. The pH was approximately 7.3, and the dissolved oxygen and ammonia concentrations were > 8 and about 0.1 mg/L, respectively. The water temperature was kept at 23.7 ± 1.2°C with heat rods. All fishes were allowed to acclimate for at least seven days and fed daily with a drug-free dry feed (pellet size 3 mm) purchased from Henan Tongwei Feed Co., Ltd. (Xinxiang, China). Animal experiments were conducted under protocols approved by the Institutional Animal Care and Use Committee (IACUC) of Henan University of Science and Technology.

### Drug Administration and Sampling

The raw material of danofloxacin mesylate was dissolved in saline to prepare an oral solution with a concentration of 5 mg/ml. Before oral dose, each fish was weighed, and the corresponding dosing volume was accurately drawn with a 1-ml syringe. Each fish was numbered and observed for 20 s after oral administration by gavage at 10 mg/kg BW. Those without emesis retained the experiment and were put back into the cuboid tank.

At 0.25, 0.5, 1, 2, 4, 6, 8, 12, 16, 24, 36, 48, 72, 96, 120, and 144 h after administration, the blood was collected from the fishtail vein, immediately placed in a test tube containing heparin sodium. According to the previous research methods (14, 17), the blood collection schedule is shown in Table 1. Each fish was sampled sparsely four times. Plasma samples were further collected by centrifugation at 2,000 × g for 10 min. All collected samples were frozen and stored at −20°C until further analysis.

**Table 1:** Subject ID, body weight, and blood collection time (hour) points from Yellow River carp after administration.

**Subject ID**	**Body weight (kg)**	**0.25**	**0.5**	**1**	**2**	**4**	**6**	**8**	**12**	**16**	**24**	**36**	**48**	**72**	**96**	**120**	**144**
1	0.40	^*^				^*^				^*^				^*^			
2	0.37		^*^				^*^				^*^				^*^		
3	0.35			^*^				^*^				^*^				^*^	
4	0.49				^*^				^*^				^*^				^*^
5	0.31	^*^				^*^				^*^				^*^			
6	0.39		^*^				^*^				^*^				^*^		
7	0.25			^*^				^*^				^*^				^*^	
8	0.41				^*^				^*^				^*^				^*^
9	0.37	^*^				^*^				^*^				^*^			
10	0.33		^*^				^*^				^*^				^*^		
11	0.37			^*^				^*^				^*^				^*^	
12	0.37				^*^				^*^				^*^				^*^
13	0.37	^*^				^*^				^*^				^*^			
14	0.32		^*^				^*^				^*^				^*^		
15	0.37			^*^				^*^				^*^				^*^	
16	0.41				^*^				^*^				^*^				^*^
17	0.29	^*^				^*^				^*^				^*^			
18	0.53		^*^				^*^				^*^				^*^		
19	0.25			^*^				^*^				^*^				^*^	
20	0.40				^*^				^*^				^*^				^*^
21	0.42	^*^				^*^				^*^				^*^			
22	0.54		^*^				^*^				^*^				^*^		
23	0.43			^*^				^*^				^*^				^*^	
24	0.37				^*^				^*^				^*^				^*^

The ^*^ symbol indicates that this individual was sampled at this time point.

### Analytical Method

The plasma sample was transferred from −20 to 4°C for thawing before treatment, and then 200 μl of plasma was spiked with the same volume of acetonitrile. After vortexing for 30 s and centrifugation at 4,000 × g for 10 min, the supernatant was evaporated to dryness with a stream of nitrogen at 60°C. The residue was redissolved in 500 μl of the mobile phase. After vortexing for 1 min and centrifugation for 10 min at 12,000 × g, the supernatant was filtered through a 0.22-μm filter into the autosampler glass vial. The supernatant (20 μl) was injected onto the C-18 column.

The Waters e2695 HPLC system with a 2,475 fluorescence detector (Waters, USA) was used to determine danofloxacin concentrations. The chromatographic column was Hypersil BDS C18 (250 × 4.6 mm inner diameter, 5 μm; Dalian Elite Analytical Instruments Co., Ltd.; Dalian, China) kept at 30°C. The mobile phase was 18% acetonitrile and 82% phosphoric acid buffer (0.05%; adjusting the pH to 2.8 with triethylamine). And its flow rate was set as 1 ml/min. The excitation and emission wavelengths of the fluorescence detector were set as 280 nm and 450 nm, respectively.

### Validation of the Analytical Method

The analytical standard of danofloxacin mesylate was dissolved in an appropriate amount of pure water and then diluted with methanol to obtain a stock solution with a concentration of 1 mg/ml (calculated as danofloxacin base) and stored at −20°C until use. Calibration standards and quality control samples were prepared by diluting the stock solution with blank plasma samples. The concentration range of the calibration curve was between 0.005 and 2 μg/ml. For precision and accuracy, five replicates at three different concentrations (0.005, 0.1, and 2 μg/ml) were tested to evaluate coefficients of variation and recoveries, respectively. For sensitivity, the limits of quantification (LOQ) and detection (LOD) were determined based on signal-to-noise ratios of ≥10 and ≥3, respectively.

### Population Pharmacokinetic Modeling

Non-compartmental analysis by naïve pooled approach was firstly performed in Phoenix (version 8.1; Pharsight, Cary, NC, USA) to determine the initial values of population pharmacokinetics analysis. Then Phoenix NLME software was used to perform the non-linear mixed-effects modeling analysis. And the base structural pharmacokinetic model was single compartment extravascular administration. This population model was continuously optimized by changing the analysis algorithm and residual error model, including additive, multiplicative, log additive, and mixed.

We evaluated the fit goodness of the population pharmacokinetic model through different diagnostic plots, such as dependent variable (DV) vs. individual predicted concentration (IPRED), as well as conditional weighted residuals (CWRES) plot. Multiplicative was selected as the residual error model through the goodness of fit of conditional weighted residuals plot. The residual error term was assumed to be normally distributed, the average value was 0, and the variance was R^2^. Finally, the first-order conditional estimation extended lead squares (FOCE-ELS) algorithm was used to analyze and estimate the population's typical values and related variation coefficients.

The role of covariates should be evaluated in population pharmacokinetic studies to determine the source of variation between individuals. Covariates refer to the typical characteristics of the research object (weight, gender, race, biochemical indexes, etc.) in population analysis. The body weight (expressed as mean) was selected as the covariate in the present study. Then its retention or removal was judged according to the −2LL (−2 × log maximum likelihood) value, the likelihood ratio test (LRT), and the Akaike's inclusion criteria (AIC). When there were two or more random effects in the model, their correlation should be considered, and the relationship should be described by adding covariance. The final model also used bootstrap to verify the stability, and the number of runs was 1,000.

Based on the model, the pharmacokinetic parameters (Ka, V, and CL) of each individual were calculated according to the following equation:


(1) $$\begin{array}{l} Kaí\;=\;tvKa\;\times \;\left(wtí/0.38\right){\;}^{\wedge }\;dKadwt\;\times \;exp\left(\text{η}Kaí\right) \end{array}$$

Where tvKa is the typical value (population mean) of absorption rate constant, the dKadwt is body weight effect on Ka, representing the variation caused by the clinical characteristics (fixed effect) of the subject (results not shown), Kaí and wtí are the Ka and body weight of the íth animal, respectively, and the average body weight of all animals is 0.38 kg. The eta (ηKa) conformes to the normal distribution, and the variance is ω^2^. Estimation ω^2^ is the inter-individual variation of the population model, which is together with the residual error of measuring drug concentration called a random effect. The other two parameters of CL and V were calculated by the same algorithm with Ka.

Secondary parameters, including elimination rate constant (K_10_), elimination half-life (T_1/2β_), and the area under the curve (AUC_0−inf_), were calculated by classical equations. The results are shown in Table 2, and the formula was defined as follows:


(2) $$\begin{array}{lll} K_{10} & = & tvCL/tvV \end{array}$$

(3) $$\begin{array}{lll} T_{1/2\text{β}} & = & ln2/K_{10} \end{array}$$

(4) $$\begin{array}{lll} AUC_{0-\text{inf}} & = & Dose/tvCL \end{array}$$

where Dose is the applied dose (10 mg/kg BW) and tvCL is the population mean value for clearance rate.

**Table 2:** Secondary parameter results calculated based on the final model (Model 3).

**Secondary parameters**	**Units**	**Mean (SE)**	**CV%**	**2.5% CI−97.5% CI**
K_10_	1/h	0.015 (0.001)	5.46	0.013 to 0.016
T_1/2β_	h	47.7 (2.61)	5.46	42.5 to 52.9
AUC_0−inf_	h·μg/ml	14.4 (0.256)	1.77	13.9 to 14.9

*K_10_, elimination rate constant; T_1/2β_, elimination half-life; AUC_0−inf_, area under the curve from zero to infinity*.

## Results

### Validation of the Analytical Method

Danofloxacin had a good correlation in the concentration range from 0.005 to 2 μg/ml. The calibration standard curve was Y = 3e−09X−0.0076 (*R*^2^ = 0.9995), where Y and X were the danofloxacin concentration and chromatographic peak area, respectively. The LOQ was determined as 0.005 μg/ml. The average extraction recovery of danofloxacin in carp plasma was 94.03% (82.2–106.91%), and the intra- and inter-day coefficients of variation ranged from 0.36 to 7.36% and from 4.74 to 7.97%, respectively (Table 3).

**Table 3:** Recovery (%) and the CV (%) results of danofloxacin in Yellow River carp plasma.

**Concentrations (μg/ml)**	**Day**	**Repeat**					**Intra-day CV%**	**Inter-day CV%**
		**1**	**2**	**3**	**4**	**5**		
0.005	1	85.87	98.46	82.20	83.62	88.53	7.36	5.97
	2	96.19	96.55	95.77	88.41	91.18	3.88	
	3	83.65	95.29	92.19	94.09	86.29	5.62	
0.1	1	98.97	103.07	106.91	106.91	106.70	3.35	7.97
	2	90.01	90.04	89.55	89.66	89.27	0.36	
	3	93.80	95.01	82.45	91.99	95.70	5.89	
2	1	100.70	90.93	95.53	100.47	99.27	4.27	4.74
	2	97.02	93.89	101.18	104.11	101.08	4.03	
	3	91.42	91.81	92.11	91.14	92.56	0.61	

### Pharmacokinetic Results

Danofloxacin was detectable within 144 h after the single oral administration at 10 mg/kg BW. Non-compartmental analysis by naïve pooled approach was performed to determine the population model's initial values, and the results are shown in Table 4. Large variability was observed for some parameters, including AUC_0−24hr_, V/F, T_max_, T_1/2β_, and C_max_.

**Table 4:** Initial values of pharmacokinetic parameters obtained by non-compartmental analysis by naïve pooled approach.

**Parameters**	**Units**	**Mean**	**SD**
AUC_0−24hr_	h·μg/ml	2.43	5.74
AUC_0−inf_	h·μg/ml	4.48	1.29
V/F	L/kg	87.3	75.5
CL/F	L/h/kg	0.771	0.295
T_max_	h	10.6	14.5
C_max_	μg/ml	0.166	0.321
T_1/2β_	h	80.3	50.2

*AUC_0−24hr_, area under the curve from zero to the twenty-four hours; AUC_0−inf_, area under the curve from zero to infinity; V/F, apparent volume of distribution per bioavailability; CL/F, total body clearance per bioavailability; T_max_, time to maximal plasma concentration; C_max_, maximum plasma concentration; T_1/2β_, terminal half-life*.

### Model Comparison

**Figure 1:**
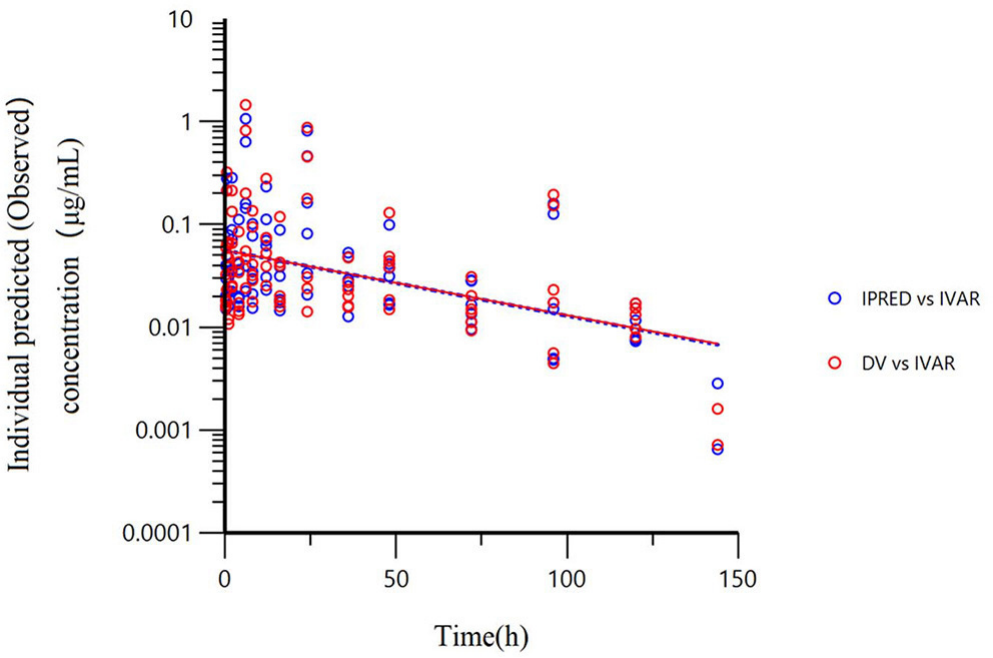
The final population model (Model 3) fitted the regression curves of individual predicted concentration and observed concentration. The final model was used to predict the concentration and regression curve after a single oral administration of 10 mg/kg BW of danofloxacin. Red dots represent the observed value; blue dots represent the predicted value; The solid line represents the actual curve and the dotted line represents the prediction curve. The independent variable (IVAR) refers to time, the dependent variable (DV) refers to observed concentration. The ordinate is logarithmic.

**Figure 2:**
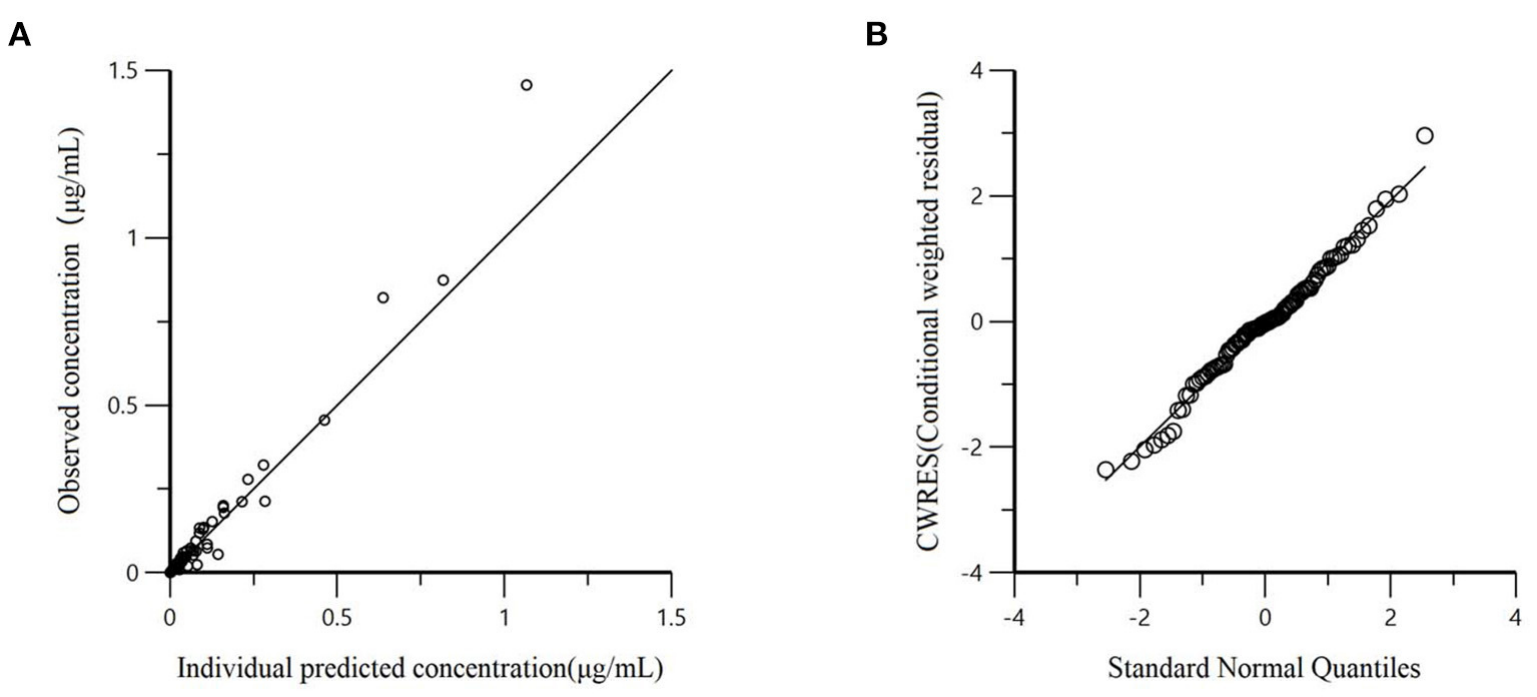
**(A)** Dependent variable (observed concentration) vs. individual predicted concentration based on final Model 3; **(B)** The conditional weighted residuals (CWRES) conform to the standard normal distribution.

All models' pharmacokinetic parameter prediction results (Table 5) were derived from non-covariates (Model 1) and covariates (Models 2 and 3). Actually, the volume of distribution (V/F) and clearance (CL/F) represented the apparent volume of distribution and clearance per fraction absorbed for an oral dose, respectively. In Model 1, the predicted values of tvV and tvCL were 59.8 L/kg and 0.578 L/h/kg, respectively; but the CV% value was enormous. Compared with Model 1, the CV% values of tvCL, tvV, and tvKa significantly decreased after adding covariates (body weight) in Model 2. The covariate model reduced the unexplained inter-individual variation, especially the absorption rate constant. Then, based on Model 2, the covariance model was added to obtain Model 3. The CV% of all pharmacokinetics parameters decreased significantly, significantly different from Model 1 and Model 2 (*P* < 0.001). The model's prediction ability was judged according to the goodness of fit of the diagnostic plots. Finally, we chose Model 3 as the final population model to calculate the corresponding pharmacokinetic parameters. Figure 1 shows the concentration (observed and predicted ones) vs. the time curve based on Model 3, indicating excellent fits for the two regression curves. The diagnostic plots of Model 3 are shown in Figure 2.

**Table 5:** Parameter estimation of population pharmacokinetic model.

**Parameter**	**Units**	**Mean (SE)**	**CV%**	**2.5% CI−97.5% CI**	**Interindividual variation ω^2^ (CV%)**	**Mean (SE) bootstrap estimates (*n* = 1,000)**
**Model 1**. No covariates model						
tvKa	1/h	2.21 (0.898)	40.6	0.427 to 4.00	2.23 (60.8)	/
tvV	L/kg	59.8 (12.3)	20.6	35.4 to 84.2	1.26 (33.4)	/
tvCL	L/h/kg	0.578 (0.151)	26.2	0.277 to 0.879	0.675 (35.9)	/
Residual error	μg/ml	0.359 (0.060)	16.6	0.241 to 0.478	/	/
**Model 2**. Covariates body weight model						
tvKa	1/h	1.89 (0.698)	37.0	0.500 to 3.28	1.48 (44.7)	/
tvV	L/kg	57.2 (10.4)	18.2	36.5 to 77.8	1.16 (34.3)	/
tvCL	L/h/kg	0.566 (0.136)	24.1	0.294 to 0.837	0.619 (35.4)	/
Residual error	μg/ml	0.360 (0.049)	13.7	0.262 to 0.459	/	/
**Model 3**. Covariates body weight and covariance model						
tvKa	1/h	2.48 (0.123)	4.94	2.24 to 2.73	3.45 (0.203)	2.97 (0.975)
tvV	L/kg	47.8 (1.90)	3.97	44.0 to 51.6	1.19 (8.40)	52.5 (9.35)
tvCL	L/h/kg	0.694 (0.012)	1.77	0.669 to 0.718	0.712 (4.35)	0.705 (0.117)
Residual error	μg/ml	0.339 (0.014)	4.06	0.312 to 0.367	/	0.337 (0.043)

*Typical value (tv) Ka, absorption rate constant; tvV, apparent volume of distribution per bioavailability; tvCL, total body clearance per bioavailability; Residual error, random error of danofloxacin concentration in carp plasma in the model; SE, the standard error for an estimate; CV%, coefficient of variation; /, not applicable*.

### Model Validation Results

The stability of the final Model 3 was verified by bootstrap and a total of 1,000 sets of new samples were generated by the random bootstrap sampling method with the original data set. The fitting results were compared between the original and new data sets. The average estimated values were similar to the final model results, and the results are shown in Table 5.

## Discussion

To our knowledge, this is the first report on the population pharmacokinetic modeling of danofloxacin in Yellow River carp. The pharmacokinetic parameters were firstly obtained for danofloxacin based on the non-compartmental analysis by naïve pooled approach. However, almost all of them had huge standard deviation (SD) values, indicating a lousy prediction ability. The non-compartmental analysis was also performed based on the average concentrations vs. time data (results not shown); however, neither SD value for each pharmacokinetic parameter nor good predictions could be obtained. Therefore, the non-compartmental analysis results (Table 4) only served as the initial values of the population pharmacokinetic model.

During constructing the population model, the values of −2LL and AIC for Model 1 (without covariates) were both smaller than Model 2 (with covariates). The choice of covariate model was the maximum likelihood ratio test (LRT), LRT = Δ-2LL, conforming to Chi-square distribution, and the calculated LRT was 7.23, which indicated that there was a significant difference between the two models (*P* < 0.01). The LRT was 35.9 between Models 2 and 3, showing a significant difference (*P* < 0.001). This significant difference might be caused by the addition of covariance. In the process of establishing the model, it was found that each eta (ηKa, ηCL, and ηV) was not independent of the others, and there was a correlation between them, which was measured by the Pearson correlation coefficient. Therefore, body weight as a covariate indeed had a certain effect on the pharmacokinetics of danofloxacin in carps. The present effect of covariates on the population model was similar to a previous study of tobramycin in horses. And it was reported that adding body weight and creatinine clearance as covariates could improve the prediction ability. In contrast, gender as another covariate did not improve the prediction ability of the model (18). In another population pharmacokinetics study in the trout, it was found that the ploidy of fish had an impact on the pharmacokinetic results of enrofloxacin (19).

In the current study, the parameters of tvV, tvKa, and T_1/2β_ calculated from the final Model 3 were 47.8 L/kg, 2.48 h^−1^, and 47.7 h, respectively. The absorption rate constant was greater than those reported in Acipenser schrenckii reared at a similar temperature (23°C) with the same route and dosage (0.329 h^−1^) (10). However, the elimination half-life was much lower than that of Acipenser schrenckii [78.267 h; (10)]. In tilapia cultured at a temperature ranging from 25 to 27°C, faster absorption and elimination were also found than the current results, and the T_1/2β_ was reported as 29 h (8). After single oral administration at 20 mg/kg BW in turbot reared at 16°C, the Ka and Vd values for danofloxacin (0.11 h^−1^; 2.986 L/kg) were smaller than the present ones in carp; however, the corresponding T_1/2β_ was prolonged to 129.2 h (20), which might be because of the different water temperatures. Within a specific temperature range, the metabolic rate of a drug in a fish is directly proportional to the water temperature. Generally, the metabolic and elimination rate will increase by 10% for every 1°C increase in water temperature (21). However, in the experiment of rainbow trout reared at 11.7°C, the T_1/2β_ of danofloxacin after single oral administration at 10 mg/kg BW was only 41 h (11), which was lower than the above warm water fish and our report. These differences might be due to the varied salinity of seawater; the previous study had proved that the metabolic rate of rainbow trout in seawater was higher than that in freshwater (22).

Many factors could affect the pharmacokinetics in aquatic animals, such as species differences, water temperature, and salinity. Danofloxacin pharmacokinetic parameters in fish showed species differences, which might be caused by physiological differences such as metabolic enzyme activity, renal function, and muscle composition (23).

Due to various physical and chemical properties, the metabolic rates vary significantly from different fluoroquinolones in fish. In common carp reared at 17°C, the T_1/2β_, AUC_0−inf_ and T_max_ of enrofloxacin following oral administration at 10 mg/kg BW were determined as 44.2 h, 117.4 h·μg/ml, and 26 h, respectively; however, the T_1/2β_ of its active metabolite ciprofloxacin was 390.5 h (24). While for norfloxacin, the Ka, T_1/2β_ and AUC_0−inf_ were reported as 2.16 h^−1^, 26.33 h, and 103.1 h·μg/ml, respectively, in common carp cultured at 20°C with the same route and dosage (25). Compared with the current results, the tvKa of danofloxacin in carp was similar to norfloxacin. However, the T_1/2β_ of danofloxacin was significantly slower than that of norfloxacin, similar to that of enrofloxacin. In addition, the area under the concentration-time curve (14.4 h·μg/ml) of danofloxacin is much lower than that of the above two drugs. Considerable differences of these three fluoroquinolones in carp might be due to the diverse water temperatures; however, further studies should be conducted to find other possible reasons.

Danofloxacin showed an earlier peak time (T_max_ = 45 min) and a shorter elimination half-life (t_1/2β_ = 15 h) in koi after intramuscular injection (13). However, intramuscular and intravenous injections are not commonly used in large-scale fish cultures because they require more time and staffing. It is only used for high-value ornamental fish like koi (26). Generally, it is more appropriate for farmers to administer the fish through a medicated feed. However, this oral administration route was not the best one in a pharmacokinetic study because it was difficult to accurately calculate the amount of drugs entering the fish due to the unknown amount of drugs dissolved in the water. Compared with intramuscular injection and oral administration, danofloxacin in bath administration showed very low bioavailability (F = 10.09%). The authors speculated that the location of drug absorption and the drug might form chelates with metals in water so as to reduce drug absorption during bath administration (11). Therefore, this study used oral administration by gavage to prevent possible drug loss due to feeding.

In this study, the non-linear mixed effect model was used to describe the population pharmacokinetics of danofloxacin in Yellow River carp. On the premise of sparse sampling, the typical values of the population (fixed effect) and inter-individual variation (random effect) were thoroughly described. Population pharmacokinetic studies have been performed in some marine or aquaculture animals, such as green sea urchin (27), purple sea stars (28), harbor seals (17), giant freshwater prawns (29), rainbow trout (19), and tilapia (30). Because population pharmacokinetics allows the determination of factors that may affect the pharmacokinetics, it is increasingly used for individualized administration to reduce the risk of inaccurate doses and the production of drug-resistant strains in animal populations (31). Population pharmacokinetics often needs to record the physiological and pathological factors of the treatment objects in practical application because considering the broad differences between the treatment objects and the continuous changes of individual plasma concentration during the treatment. These factors are the typical characteristics of the treatment object and can be incorporated into the population model as covariates to explain some differences among individuals. These characteristics (age, gender, body weight, creatinine clearance, and infection status) are obviously helpful in establishing individual drug dosing regimens. It was challenging to find these characteristics in healthy carp in the current study. Therefore, only body weight was finally recorded and included in the covariate model. In addition, because only healthy carp were used in the present study, the infection was not taken into consideration. Therefore, our study did not obtain more individual characteristics (such as renal function) to explain the variation between individuals further. However, it is a meaningful attempt to get more accurate pharmacokinetic parameters using fewer experimental animals.

As concentration-dependent antibacterials, the PK/PD indices for fluoroquinolones have been proved as AUC/MIC ratio ≥ 125 and C_max_/MIC ratio ≥ 10 to provide clinical and bacteriological success and prevent the emergence of resistance (32). When the water temperature was 25°C, the MIC value of danofloxacin against *Yersinia ruckeri* isolated from rainbow trout was determined to be 0.016 μg/ml (11). However, the MIC data of danofloxacin was not available against any pathogens isolated from Yellow River carp. Assuming danofloxacin has the same MIC values against Yellow River carp pathogens, the calculated AUC/MIC and C_max_/MIC ratios were 900 and 10.4, respectively. Therefore, the current 10 mg/kg danofloxacin oral administration can provide suitable plasma concentrations to inhibit those pathogens with MIC values below 0.016 μg/ml. The abundant and accurate aquaculture species-specific MIC data could successfully predict the clinical outcome of antimicrobial treatment. Therefore, the danofloxacin MIC data against Yellow River carp-specific pathogens should be further collected to draw a reasonable dosing regimen.

## Conclusion

In the present study, danofloxacin indicated favorable pharmacokinetic properties, including slow absorption and elimination in Yellow River carp. The sparse sampling method accompanied by the non-linear mixed effect modeling method was validated to predict the pharmacokinetics of danofloxacin in Yellow River carp. After adding the covariate (body weight) and covariance model, the coefficient of variation of pharmacokinetic parameters and the variation between individuals decreased significantly. The current danofloxacin oral dosing (10 mg/kg BW) can provide suitable concentrations to inhibit those pathogens with MIC values below 0.016 μg/ml based on the calculated ratios of AUC/MIC or C_max_/MIC. Further studies are still needed to determine the *in vitro* and *in vivo* antibacterial efficacy of danofloxacin against pathogens isolated from Yellow River carp and finally draw a reasonable dosing regimen.

## Data Availability Statement

The raw data supporting the conclusions of this article will be made available by the authors, without undue reservation.

## Ethics Statement

The animal study was reviewed and approved by the Institutional Animal Care and Use Committee (IACUC) of Henan University of Science and Technology.

## Author Contributions

FanY conceived this project. Z-WS, YD, and C-SZ performed the pharmacokinetics experiments. FangY, H-TS, K-LM, and Z-EL determined the danofloxacin concentrations in collected samples. FangY and HW performed the pharmacokinetic analysis. Z-WS wrote this manuscript with support from FangY. All authors have read and approved this final manuscript.

## Funding

This research was funded by the National Natural Science Foundation of China (Grant No. 31402253) and the Foundation for University Young Key Teacher Program of Henan Province (Grant No. 2021GGJS044).

## Conflict of Interest

The authors declare that the research was conducted in the absence of any commercial or financial relationships that could be construed as a potential conflict of interest.

## Publisher's Note

All claims expressed in this article are solely those of the authors and do not necessarily represent those of their affiliated organizations, or those of the publisher, the editors and the reviewers. Any product that may be evaluated in this article, or claim that may be made by its manufacturer, is not guaranteed or endorsed by the publisher.
